# The effect of swimming on the body posture, range of motion and musculoskeletal pain in elite para and able-bodied swimmers

**DOI:** 10.1186/s13102-023-00734-z

**Published:** 2023-09-25

**Authors:** Anna Zwierzchowska, Eliza Gawel, Jakub Karpinski, Adam Maszczyk, Aleksandra Zebrowska

**Affiliations:** Institute of Sport Sciences, The Jerzy Kukuczka Academy of Physical Education in Katowice, Mikołowska 72A, Katowice, 40-065 Poland

**Keywords:** Low back pain, Spine, Paralympic sport, Athletes with disabilities, Compensatory mechanisms, Sports training, Shoulder pain, Adaptation, Pelvic inclination

## Abstract

**Background:**

Elite swimmers may be predisposed to disturbances in the range of motion (ROM) of hip joints and spinal curvatures, which are a factor that induces body’s compensatory mechanisms that may have an impact on sports training, athletic performance and health. This study aimed to identify compensatory mechanisms in body posture of elite Para and able-bodied swimmers (spinal curvatures, ROM), to indicate the dominant locations of the compensatory mechanisms in the groups of Para and able-bodied athletes, and to identify and compare the prevalence and location of musculoskeletal pain from the last week and the last six months in the context of compensatory mechanisms.

**Methods:**

Thirty-five (nF = 8; nM = 27; age = 20.51 ± 4.24) elite Para and able-bodied swimmers from the Polish national team took part in the study and were divided into: study group (SG) of Para swimmers and control group (CG) of able-bodied swimmers. Depth of the anteroposterior spinal curvatures and sagittal spinal mobility testing were evaluated with a Medi Mouse device. The prevalence and locations of musculoskeletal pain were assessed with a Nordic Musculoskeletal Questionnaire for the last seven days (NMQ-7) and the last six months (NMQ-6).

**Results:**

In both groups lumbar hypolordosis, anterior pelvic tilt and pain in the shoulders, lower back and hips/thighs (NMQ-7) were reported the most frequent. In SG several significant relationships were found between duration of sport-specific training experience (years) and depth of angle the lumbar lordosis, the depth of the angle of pelvic inclination the ROM in the lumbar spine and thoracic spine, what was not reported in CG.

**Conclusions:**

Extrinsic compensatory mechanism was identified in both study groups, however only in SG it occurred as structural (depth of the angle of lumbar lordosis and pelvic inclination) and functional changes (ROM in the thoracic and lumbar spine) in the body posture. Internal compensatory mechanism was identified in SG, however external compensation showed only partially suppressive character regarding to internal compensation. The locations of the musculoskeletal complaints seems to result from both internal compensatory mechanism (SG) and continuous overload of the anatomy trains as a result of swimming training (SG, CG).

## Background

Swimming is known as a complete sport based on four main strokes (freestyle, butterfly, breaststroke, and backstroke) and involves the coordination of the upper and lower limbs to propel the body forward in a non-weight-bearing environment [1]. The majority of propulsive forces are generated by repeated overhead movements of the upper limbs (85–90%), with only small contribution (10–15%) from lower limbs [2, 3]. However, lower limb work and core strength are significant for neutralizing the body’s reaction to the upper limb actions by diminishing trunk inclination [3, 4]. In competitive swimming at an elite level, regardless of the stroke specialization, swimmers spend most of the training volume swimming freestyle [5] which is characterized by repeated movements of the upper and lower limbs, potentially leading to an overload of the musculoskeletal structures, especially in the upper limbs and spine [5, 6]. Furthermore, elite swimmers may be predisposed to disturbances in the range of motion (ROM) of hip joints and spinal curvatures, associated with more frequent musculoskeletal complaints and decreased swimming performance [7].

The ability to perform effortless and pain-free movements with proper muscle and joint function is strictly related to correct body posture [8]. Disturbances in the balance of musculoskeletal structures are a factor that impacts human posture, inducing the body’s compensatory mechanisms [9]. A meta-analysis conducted by Zwierzchowska et al. [10] revealed two models of compensatory mechanisms: internal and external. The model of internal compensation is observed especially in people with mobility impairments, whose proper musculoskeletal function is limited or lost due to congenital or acquired disability. It is considered an intrinsic pathobiomechanical phenomenon in which activation of the compensatory reserves occurs unconsciously as an orthostatic response when the basic mechanisms responsible for the musculoskeletal function are disturbed [10]. In this model, the human body aims to restore musculoskeletal function (partially or fully) and keep an upright position by using specific countermeasures (musculoskeletal structures). On the other hand, the mechanism of external compensation can occur both in able-bodied athletes and those with disabilities as it is defined as the adaptation of musculoskeletal structures to the specificity of sports technique resulting in pathobiomechanical compensations in musculoskeletal structures [9, 10]. In this case, the locomotor system in a Para athlete has to adapt to the disturbed motor function and the intrinsic internal compensatory mechanism that, combined with high training volume and intensity, may lead to deepening of the patobiomechanical changes in the musculoskeletal system and inducing musculoskeletal disorders.

In the available scientific literature, few studies have examined sagittal spinal curvatures or ROM in able-bodied swimmers [7, 11–13]. Furthermore, to the best of our knowledge, only one study addressed this issue in Para swimmers [14]. Moreover, there is still a lack of research on how to optimize sports training of Para and able-bodied swimmers in the aspects of prevention of long-term exclusion from competing at an elite level. A meta-analysis of the studies from the last decade that have assessed the body posture of competitive athletes indicated that the overall rates of postural disturbances in elite athletes are high and may be associated with compensatory mechanisms [10]. However, it is difficult to find studies that have assessed this issue in able-bodied and/or Para swimmers in the context of internal and external compensatory mechanisms and musculoskeletal pain.

Given the above and the gap in the available scientific literature, it seems reasonable to indicate the importance of compensatory mechanisms as a significant factor that may have an impact on sports training, athletic performance (musculoskeletal complaints), and athlete’s health (long-term pathobiomechanical changes in musculoskeletal structures). Accordingly, this study aimed to (1) identify compensatory mechanisms in body posture of elite Para and able-bodied swimmers (spinal curvatures, ROM), (2) indicate the dominant locations of the compensatory mechanisms in the groups of Para and able-bodied athletes, and (3) identify the prevalence and location of musculoskeletal pain from the last week and the last six months in the context of compensatory mechanisms. It was assumed that the mechanism of external compensation occurs in both Para and able-bodied swimmers whereas internal compensation is suppressed by external compensation in Para swimmers at an elite level. We have also hypothesized that the body’s extrinsic compensatory mechanisms may be related to the prevalence and locations of musculoskeletal pain.

## Methods

### Participants

Thirty-five (female = 8; male = 27; age = 20.51 ± 4.24; body mass = 69.32 ± 13.08; body height = 1.78 ± 0.12) elite Para and able-bodied swimmers from the Polish national team took part in the study. The inclusion criteria were as follows: (1) elite Para or able-bodied male or female athletes, (2) at least 4 years of training experience at an elite level, (3) at least 3 sport-specific training sessions per week, (4) satisfactory self-reported health status, (5) at least minimal disability (MD) according to the World Para Swimming classification (only for Para swimmers), (6) congenital impairment. The exclusion criteria were as follows: (1) non-elite level Para and able-bodied swimmers (2) the occurrence of an injury in the last two weeks, (3) acquired impairment, (4) withdrawal from the study. Participants were divided into two study groups i.e. study group (SG) of Para swimmers (n = 22; female = 7; male = 15) and control group( CG) of able-bodied swimmers (n = 13; female = 1, male = 12). Furthermore, athletes from the SG group were divided into the following groups based on the medical classification: blind athletes (27%), amputees (5%), Les Autres (50%), and celebral palsy athletes (18%). The majority of Para swimmers did not use any supportive equipment (n = 14) in everyday life. However, some of them used a white stick (n = 2, blinds), prosthesis (n = 1, amputees), wheelchair (n = 1, Les Autres), or walking frame (n = 1, Les Autres). Table 1 provides a detailed description of the study participants. Furthermore, both Para and able-bodied swimmers (Paralympic and Olympic Polish National Team) were in the same training macrocycle.

**Table 1:** The descriptive statistics and frequency tables of the study participants

Variables	SG Para swimmers (n = 22; nF = 7, nM = 15)	SG (males vs. females)	CG Able-bodied swimmers (n = 13; nF = 1; nM = 12)
	Mean ± SD	P value	Mean ± SD
Age (years)	19.68 ± 4.9	0.35	21.92 ± 2.43
Body Mass (kg)	63.25 ± 11.2	0.05	79.59 ± 9.15
Body Height (m)	1.73 ± 0.1	0.04	186 ± 0.07
Hip Circumference (cm)	93.02 ± 5.5	0.62	100.12 ± 3.97
Trunk fat mass (%)	18.96 ± 6.6	0.01	13.75 ± 4.19
Waist Circumference (cm)	75.8 ± 7.6	0.001	81.65 ± 4.06
BMI	21.01 ± 2.0	0.18	23.01 ± 1.79
BAI (%)	23.04 ± 4.0	0.04	21.58 ± 1.89
VFR (1–36)	4.33 ± 2.66	0.04	4.58 ± 1.44
Duration of sport-specific training experience (years)	8.64 ± 4.1	0.95	12.08 ± 3.55
Number of training sessions per week	7.76 ± 2.80	0.47	9.77 ± 1.24
Duration of disability (years)	18.73 ± 5.3	0.76	NA

n-total number of participants; nF- number of females; nM- number of males; SD – standard deviation; BMI – body mass index; BAI – body adiposity index; VFR – visceral fat rate; NA- not applicable

All measurements were carried out at The Jerzy Kukuczka Academy of Physical Education in Katowice, Poland. Study participants were allowed to withdraw from the experiment at any time and were informed about the benefits and potential risks of the study. Informed consent was obtained from all the participants and from legal guardians of the participants who were below 16 years of old. Furthermore, study participants were instructed to maintain their normal dietary and sleeping habits for 24 h before the examination. The research protocol was approved by the Bioethics Committee for Scientific Research The Jerzy Kukuczka Academy of Physical Education in Katowice, Poland (No. 9/2012) and met the ethical standards of the Declaration of Helsinki, 2013.

### Procedures

Study participants arrived at the certified Laboratory of the Diagnostics of the Body Posture and Composition (the Jerzy Kukuczka Academy of Physical Education in Katowice, Poland) in the morning (8–11 a.m.). At first, the anthropometric measurements were performed using a standard procedure and research tools similar to our previous studies [15] and included the following qualities and indicators of the body build and posture: body height (BH), body mass (BM), hip circumference (HC), waist circumference (WC), body mass index (BMI), body adiposity index (BAI), trunk fat mass, and visceral fat rate (VFR) (Tanita Viscan AB-140 Abdominal Fat Analyzer). Next, the prevalence and locations of musculoskeletal pain were assessed using a procedure proposed by Kuorinka et al. [16] with a subjective Nordic Musculoskeletal Questionnaire for the last seven days (NMQ-7) and the last six months (NMQ-6). NMQ is known to have 80–100% validity and 78–100% reliability [16]. Furthermore, to minimalize the subjective risk of error, participants received verbal instructions regarding NMQ and were instructed not to report phantom pain (SG) because of psychological factors that affect this phenomenon [17]. The questionnaire was completed in the presence of an experienced physiotherapist. Next, study participants were informed about the standardized testing procedure regarding body posture, with a visual demonstration of the spinal curvatures and spinal mobility tests in the sagittal plane.

### Sagittal spinal curvatures and spinal mobility testing

Depth of the anteroposterior spinal curvatures and sagittal spinal mobility were evaluated with a non-invasible method using a hand-held, computer-assisted Medi Mouse device (IDIAG M360) [15] that ensures reproducibility even if different researchers conduct the examinations. According to the Medi Mouse protocol, the measurement started with placing the Medi Mouse at the C_7_ level and finished at the S_3_ level. Before testing, C_7_ and S_3_ were identified by palpation and marked on the skin. Furthermore, all participants performed one practice trial on each of the testing positions to minimize the risk of error i.e., sagittal standing (arms in the habitual position), maximal sagittal standing flexion (arms straight) and maximal sagittal standing extension (arms crossed on the shoulders, elbows up). All measurements were performed by the same researcher with expertise in the measurement of spinal curvatures (EG) with randomized order of participants. All testing results were automatically recorded on a computer with Idiag M360 software immediately after finishing the examination, providing the following data in each of the three testing positions: (1) the current depth of the anteroposterior spinal curvatures (thoracic kyphosis, lumbar lordosis) and pelvic inclination and the current spinal length, (2) their normative physiological values (based on the able-bodied population of the same age, gender, body height, and body mass), (3) the differences between the current and normative physiological values of thoracic kyphosis, lumbar lordosis, pelvic inclination, and spinal length, (4) the type of sagittal spinal deviation (thoracic hyper/hypo kyphosis, lumbar hyper/hypo lordosis, anterior/posterior pelvic tilt), (5) range of motion of thoracic/lumbar segment of the spine and hip joints. The literature review of reliability and variability of Medi Mouse measurements showed ICC = 0.85 for spinal mobility measurements in the sagittal plane [18] and ICC = 0.87 for the measurements of the depth of thoracic kyphosis and ICC = 0.88 for lumbar lordosis in a sagittal standing position [19]. Furthermore, Medi Mouse has been shown to have acceptable validity [20].

### Statistical analysis

All statistical analyses were performed with the jamovi (version 2.2) computer software. The sample size was calculated using the following formula: fpc = sqrt((N-n)/(N-1)), where (i) fpc is the finite population correction factor, (ii) N is the population size, (iii) n is the sample size. Distributions, homogeneity, means and standard deviations (SD) of the anthropometric variables, participant’s characteristics (age, duration of disability, number of trainings per week, duration of sport-specific training experience (years))) and spinal curvatures (kTH, kLL, kPI, SL) were verified (Shapiro-Wilk test, Leven’s test). Due the normal distribution relationships between the analyzed variables were computed with Pearson’s correlation. Spearman’s rank-order correlation was used to calculate the correlations between the qualitative variables (NMQ-7/6) and participant’s characteristics and spinal curvatures. A comparison of the means between the studied groups was verified with a two-tailed t-test for independent samples. To determine the statistically significant relationships between the categorical variables (NMQ-7/6, qualitative characteristic of ROM and body posture) a Chi^2^ test of independence was performed. Correlations were evaluated as follows: trivial (0.0–0.09), small (0.10–0.29), moderate (0.30–0.49), large (0.50–0.69), very large (0.70–0.89), nearly perfect (0.90–0.99), and perfect (1.0) [21].

## Results

Somatic characteristics of the studied groups was assessed based on a two-tailed t-test for independent samples which showed that the spinal length of elite Para swimmers (SG) was significantly shorter compared to elite able-bodied swimmers (CG), on average 73.99 cm. This difference was statistically significant at p < 0.001, effect size 2.19 (very large). Moreover, the abovementioned t-test indicated that elite Para swimmers had significantly lower body mass than elite able-bodied swimmers, on average 16.34 kg. This difference was statistically significant at p < 0.001, effect size 1.42 (large).

**Fig. 1:**
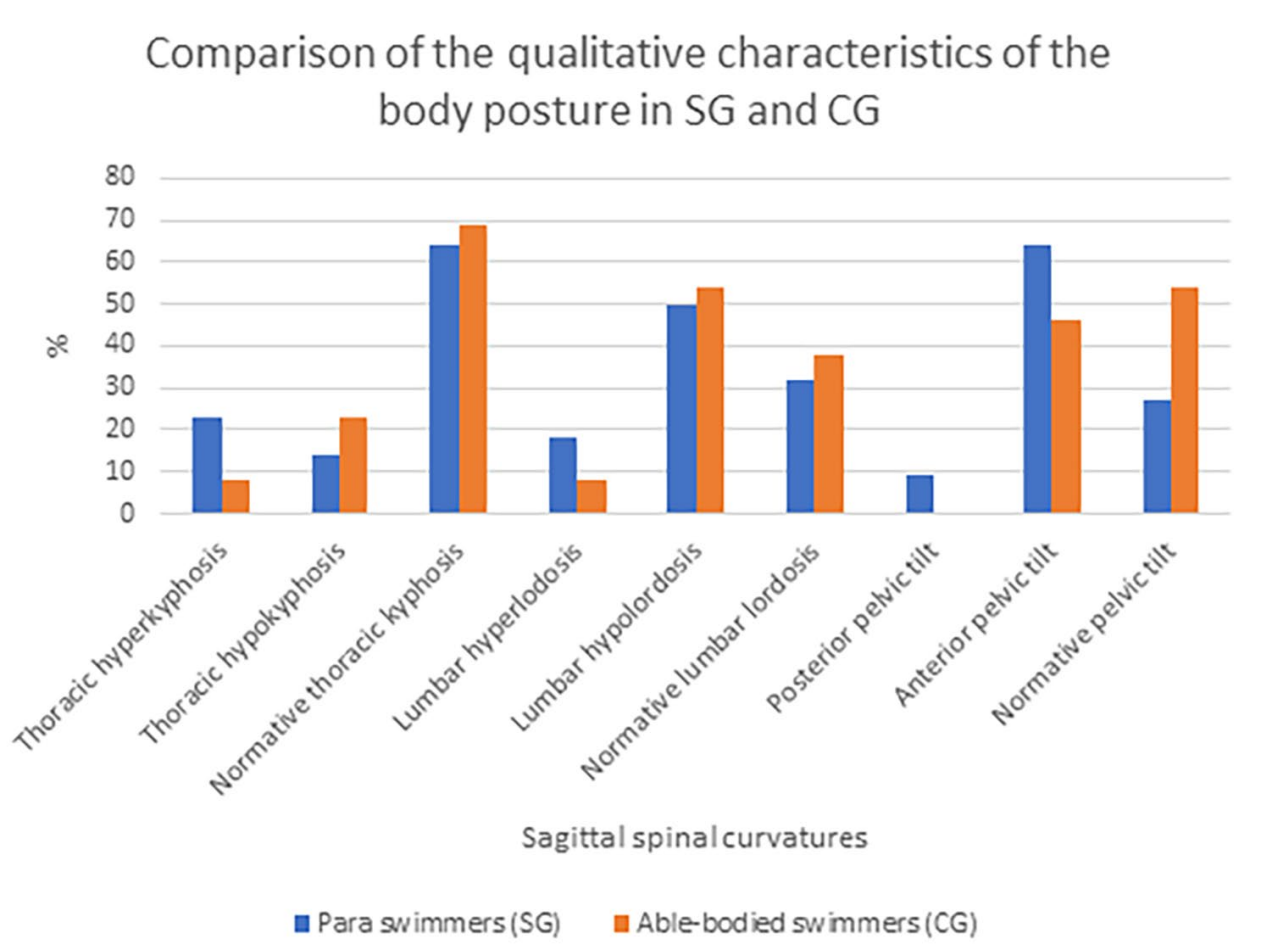
The comparison of the prevalence of the qualitative characteristics of the body posture in elite Para swimmers (SG) and elite able-bodied swimmers (CG) based on the Medi Mouse (IDIAG M-360) assessment

The results of the comparison of the qualitative (Fig. 1) and quantitative (Table 2) characteristics of the studied groups is presented below. Among SG lumbar hypolordosis and anterior pelvic tilt were the predominant postural deviations. Similar results were reported in CG. However, in both studied groups thoracic kyphosis showed mostly normative values.

**Table 2:** The descriptive statistics of quantitative characteristics of physiological spinal curvatures, pelvic inclination, and spinal length in the sagittal plane in three positions (sagittal standing, sagittal standing flexion, sagittal standing extension) in elite Para swimmers (SG) and elite able-bodied swimmers (CG).

Spinal curvatures measurements: sagittal plane	SG (n = 22) Mean ± SD (°)	SG (males and females) P value	CG (n = 13) Mean ± SD (°)	Elite Para swimmers (SG)	
				**Correlation**	R-value (p < 0.001***, p < 0.01**, p < 0.05*)
kTH-sagittal standing	42.09 ± 10.76	0.29	39.62 ± 11.21	kLL & Age (years)	R=(-0.4)*
kTH-sagittal standing flexion	51.73 ± 11.70	0.91	54.85 ± 14.92	kLL & Duration in disability (years)	R= (-0.5)**
kTH-sagittal standing extension	32.14 ± 15.72	0.27	38.38 ± 12.63	kLL & Duration of sport-specific training experience (years)	R=(-0.5)*
kLL-sagittal standing	21.81 ± 11.64	0.6	21.23 ± 8.63	kPI & Age (years)	R= (-0.4)*
kLL -sagittal standing flexion	32.55 ± 11.25	0.6	40.62 ± 8.21	kPI & Duration of sport-specific training experience (years)	R=(-0.5)*
kLL -sagittal standing extension	33.59 ± 13.80	0.27	38.85 ± 12.8	ROM in the sagittal plane (thoracic spine) & Duration of sport-specific training experience (years)	R = 0.6**
kPI -sagittal standing SL – sagittal standing	0.18 ± 1.79 472.55 ± 37.85	0.95 0.27	1.31 ± 2.18 546.54 ± 25.22	ROM in the sagittal plane (thoracic spine) & ROM in the sagittal plane (lumbar spine)	R=(− 0.5)*
kPI – sagittal standing flexion SL – sagittal standing flexion	106.05 ± 14.31 570.55 ± 38.53	0.21 0.01	108.08 ± 8.19 648.08 ± 45.62	Elite able-bodied swimmers (CG)	
kPI-sagittal standing extension SL – sagittal standing extension	25.31 ± 18.61 448.50 ± 45.44	0.67 0.13	34.85 ± 8.49 503.54 ± 27.28	**Correlation**	R-value (p < 0.001***, p < 0.01**, p < 0.05*)
ROM in the sagittal plane (thoracic spine)	20.3 ± 12.9	0.13	26 ± 38	ROM in the sagittal plane (thoracic spine) & kTH	R= (-0.6)*
ROM in the sagittal plane (lumbar spine)	62.7 ± 23.1	0.27	79.5 ± 11.2		
ROM in the sagittal plane (pelvic inclination)	135 ± 23.2	0.79	143 ± 11.6		

*kTH – thoracic kyphosis angle in the sagittal standing; kLL – lumbar lordosis angle in the sagittal standing; kPI – pelvic inclination angle in the sagittal standing ;SL – spinal length in the sagittal standing; SD – standard deviation; ROM – range of motion*

The Pearson’s correlation between the quantitative characteristics of physiological spinal curvatures, pelvic inclination and participant’s characteristics showed several significant relationships (see Table 2). According to elite Para swimmers the statistical analysis demonstrated a tendency to interpretation of the relationships between the angles of the spinal curvatures and both internal (age, duration in disability) and external variables (duration of sport-specific training experience (years)). However, such tendency was not reported in elite able-bodied swimmers.

**Fig. 2:**
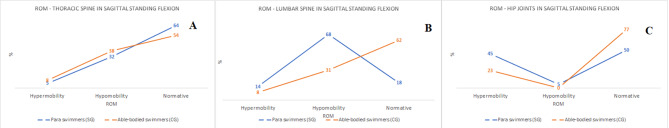
The comparison of the prevalence of ROM between SG and CG in A – thoracic spine, B – lumbar spine, C – hip joints in sagittal standing flexion (qualitative assessment)

**Fig. 3:**
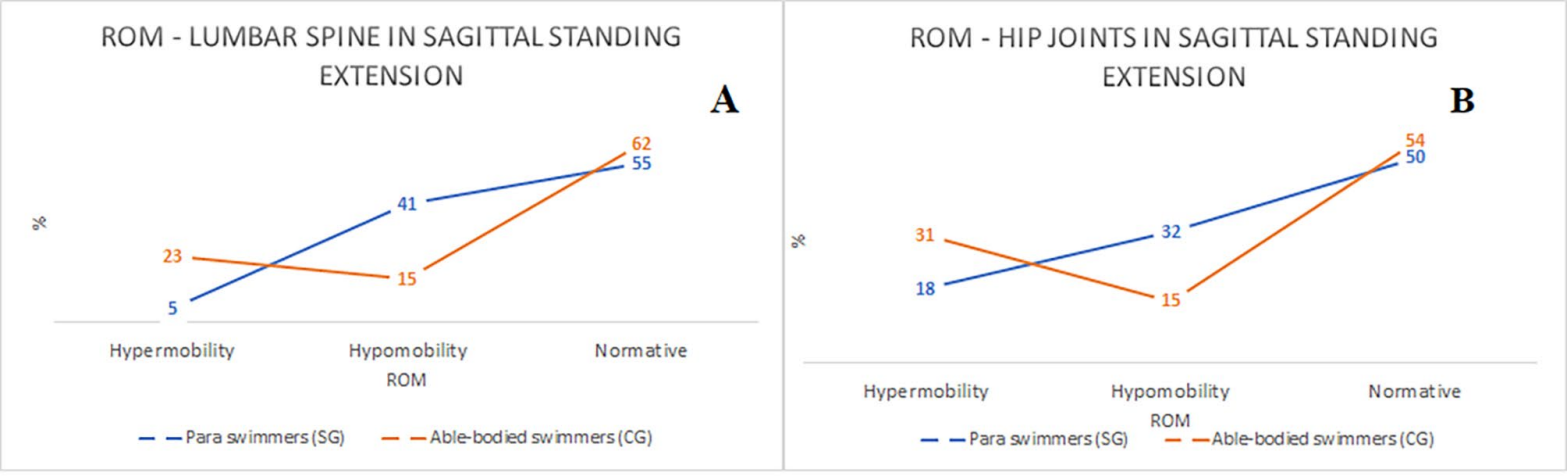
The comparison of the prevalence of ROM between SG and CG in A – lumbar spine, B – hip joints in sagittal standing extension (qualitative assessment)

The comparison of ROM based on the Medi Mouse (IDIAG M-360) assessment between the analyzed groups is presented in Fig. 2 (sagittal standing flexion) and Fig. 3 (sagittal standing extension). In both groups thoracic spine showed mostly normative values in the sagittal standing flexion, however a tendency for lumbar hypomobility and hip joints hypermobility was reported in SG. According to the sagittal standing extension a tendency for lumbar hypomobility was observed in SG which was not reported in CG..

**Fig. 4:**
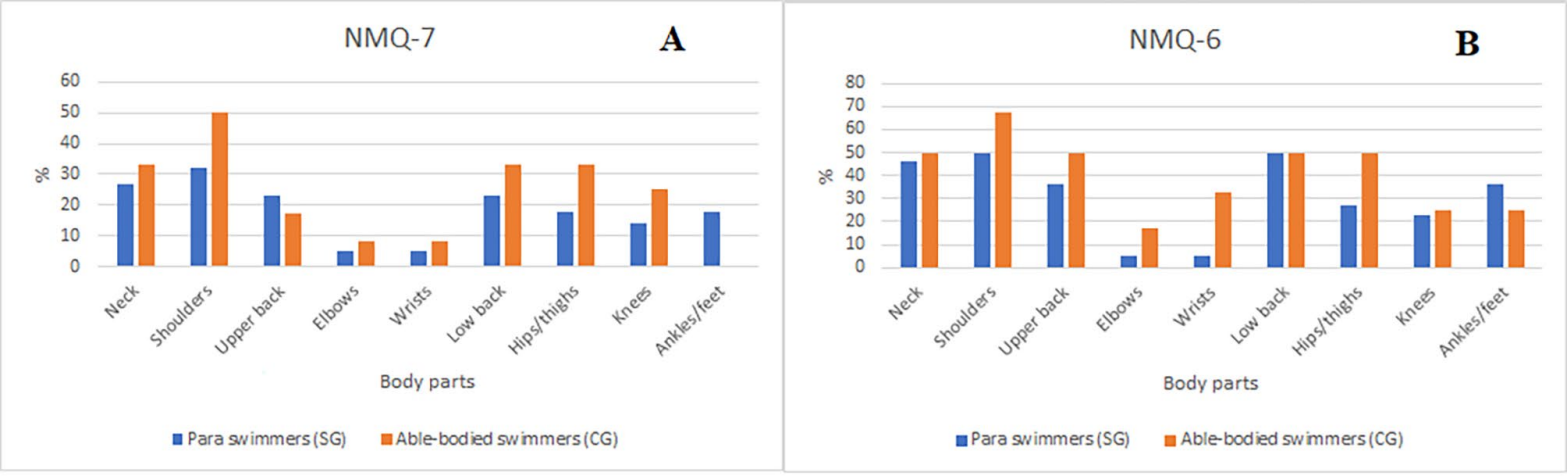
The comparison of the prevalence and location of musculoskeletal pain based on the NMQ-7 (A) and NMQ-6 (B) in SG and CG (qualitative assessment)

Figure 4 presents the comparison of the prevalence and location of musculoskeletal pain based on the NMQ-7 (A) and NMQ-6 (B) in the studied groups. According to the NMQ-7 in both groups shoulders, low back and hips/thighs were reported the most frequent. However, based on the NMQ-6 in SG neck, shoulders, lower back and ankles/feet were the most indicated painful areas, whereas in CG athletes mostly reported the following body parts: neck, shoulders, upper back, lower back and hips/thighs.

The Spearman’s rank-order correlation between the qualitative characteristics of the body posture (ROM - spinal curvatures, hip joints), musculoskeletal pain (NMQ-7/6), participant’s characteristics and quantitative characteristics of the body posture (kLL, kPI, ROM in the sagittal plane) is presented in Table 3. In SG both internal (age, duration in disability (years) and external (number of training sessions per week) variables showed statistically significant relationships with the prevalence and location of musculoskeletal pain based on the NMQ-7/6. Similarly, in CG some external variables (number of training sessions per week, duration of sport-specific training experience (years)) had statistical relationships with the NMQ-7/6.

**Table 3:** Spearman’s rank-order correlation – qualitative assessment of the body posture (ROM - spinal curvatures, hip joints), musculoskeletal pain (NMQ-7/6), participant’s characteristics and quantitative characteristics of body posture (kLL, kPI, ROM – the sagittal plane)

Elite Para swimmers (SG)		Elite able-bodied swimmers (CG)	
Correlation	R-value (p < 0.001***, p < 0.01**, p < 0.05*)	**Correlation**	R-value (p < 0.001***, p < 0.01**, p < 0.05*)
Lumbar spine – sagittal standing flexion (normative) & Duration in disability (years)	R=(-0.5)*	Hip joints – sagittal standing extension (normative) & Age	R = 0.6*
Lumbar spine – sagittal standing flexion (hypomobility) & Duration in disability (years)	R = 0.6**	Hip joints – sagittal standing extension (normative) & Duration of sport-specific training experience (years)	R = 0.6*
NMQ-7 (Shoulders) & Age	R= (-0.5)*	NMQ-7 (Elbows) & Number of training sessions per week	R=(-0.6)*
NMQ-7(Shoulders)& Duration in disability (years)	R= (-0.5)*	NMQ-7 (Wrists) & Number of training sessions per week	R=(-0.6)*
NMQ-7 (Knees) & ROM in the sagittal plane (thoracic spine)	R= (-0.5)*	NMQ-6 (Low back) & kTH	R=(-0.6)*
NMQ-6 (Upper back) & Duration in disability (years)	R=(-0.4)*	NMQ-6 (Hips/thighs) & kTH	R=(-0.7)**
NMQ-6 (Ankles/Feet) & Number of training sessions per week	R = 0.4*	NMQ-6 (Ankles/Feet) & kPI	R = 0.6*
NMQ-6 (Ankles/Feet) & kLL	R= (-0.5)*		
NMQ-6 (Hips/thighs) & kLL	R = 0.6**		
NMQ-6 (Hips/thighs) & ROM in the sagittal plane (thoracic spine)	R=(-0.4)*		
NMQ-6 (Ankles/Feet) & ROM in the sagittal plane (pelvic inclination)	R = 0.4*		

*NMQ-7/6- the Nordic Musculoskeletal Questionnaire form the last 7 days/6 months, kTH – thoracic kyphosis angle in the sagittal standing; kLL – lumbar lordosis angle in the sagittal standing; kPI – pelvic inclination angle in the sagittal standing*

A Chi^2^ test was performed between the different locations of musculoskeletal pain (NMQ-7/6). In SG there was a statistically significant relationship between LBP (NMQ-7) and shoulders pain (NMQ-7), X^2^ (1) = 13.9, p < 0.001, Cramer’s V = 0.79 and between LBP and pain in hips/thighs (NMQ-7), X^2^ (1) = 16.6, p < 0.001, Cramer’s V = 0.87. Moreover, in CG statistically significant relationships were reported i.e., between pain in the upper back (NMQ-7) and elbows (NMQ-7) and between pain in the elbows (NMQ-7) and wrists (NMQ-7), X^2^ (1) = 12.0, p < 0.001, Cramer’s V = 1. Simultaneously, in both groups no statistically relationships between NMQ-7/6 and the qualitative variables of characteristics of the body posture and ROM were reported.

## Discussion

The main finding of this study was that the external compensatory mechanisms (duration of sport-specific training experience (years)) occurred in both groups, thus confirming our initial hypothesis. However, only in elite Para swimmers the mechanism appeared as structural changes in the depth of the angle of lumbar lordosis (R=(-0.5), p < 0.05)), the depth of the angle of pelvic inclination (R=(-0.5), p < 0.05)), the ROM in the thoracic spine (R = 0.6, p < 0.01) and the lumbar spine (R=(-0.5), p < 0.05)) (see Tables 2 and 3). In contrary to SG, in elite able-bodied swimmers the external compensatory mechanism showed positive impact on the ROM in hip joints (R = 0.6, p < 0.05) (see Table 2). Moreover, the mechanism of internal compensation was identified in Para swimmers, however, external compensation showed only partially suppressive character regarding to internal compensation, as the identified relationship between the angle of lumbar lordosis and duration of disability (R=(-0.5), p < 0.01)) was much stronger than with the duration of sport-specific training experience (years) (R=(-0.5), p < 0.05)), what is partially in opposition to our initial hypothesis.

The abovementioned results confirm the two-way impact of the compensatory mechanisms on structural changes in the body posture of elite Para swimmers. Simultaneously, the results indicated that elite level swimming training in Para swimmers may suppress the disadvantageous impact of the internal compensation, while in able-bodied swimmers it may positively impact the ROM of the spinal curvatures and hip joints.

However, the results of our study are in opposition to the findings of Zaina et al. [13], who suggested that swimming training increases the risk of thoracic hyperkyphosis and lumbar hyperlordosis in able-bodied athletes. Similar conclusions were indicated in the studies of Hibbert et al. [22], who found that in competitive swimming, high training volume leads to an increase in the depth of thoracic kyphosis. Even though our study showed the occurrence of the abovementioned postural disturbances in both studied groups, most Para and able-bodied swimmers had lumbar hypolordosis and normative values of thoracic kyphosis, with only a small percentage of increased depth of thoracic kyphosis, especially in able-bodied swimmers. Nevertheless, in Para swimmers, our findings showed significant relationships between the duration of swimming training and decreased depth of the angle of lumbar lordosis in the sagittal standing regardless of the type of impairment, indicating, similar to Zaina et al. [13] and Hibber et al. [22], that swimming training impacts on the body posture of competitive athletes due to adaptative and compensatory changes in the musculoskeletal structures.

The findings of the presented study are in line with the research by Botha et al. [11], who suggested that swimming technique and long-term swimming training can induce postural disturbances in competitive swimmers. Even though Para swimmers had different types of disabilities, each impairment was congenital and they followed the same training macrocycle. Thus, it can be postulated that the external compensatory mechanism partially suppressed the impact of internal compensation on the anteroposterior spinal curvatures in elite Para swimmers.

For the issue of compensatory mechanisms in elite swimmers, our results indicated a statistically significant relationship between an external variable (duration of sport-specific training experience (years)) and normative ROM in the hip joints (able-bodied swimmers) and greater ROM in the thoracic spine (Para swimmers) (see Tables 2 and 3). Simultaneously, some relationships between an internal variable (duration of disability) and reduced ROM in the lumbar spine were reported in Para swimmers (see Table 3). Given the obtained results is may be postulated that a disturbance of body’s biomechanics, as a result of a motor impairment, is the crucial factor that may impact on both the prevalence and strength of the compensatory mechanisms in Para swimmers. At the same time, swimming training does not seem to impact on the changes of the ROM of the spinal curvatures and hip joints in elite able-bodied swimmers. Unfortunately, in the current literature it is difficult to find a single study that had examined ROM of the spinal curvatures in swimmers. Nevertheless, Shimura et al. [14] suggested that impairment of an upper limb may restrict ROM in shoulder flexion and cause excessive lumbar lordosis, because of body’s compensatory movements. In this study, the majority of Para swimmers had lower limb impairment, decreased ROM of the lumbar spine and decreased angle of the lumbar lordosis, what similar to Shimura et al. [14], indicate on body’s compensatory mechanisms.

It can not be directly indicated which compensatory mechanism was responsible for the prevalence and location of the musculoskeletal pain, especially in elite Para swimmers. However, based on the studies by other authors [6, 7, 13, 23] and the results of the statistical analyses (Chi^2^ test, Spearman’s correlation) it can be speculated that the locations of the musculoskeletal complaints are result from both internal compensatory mechanism (Para swimmers) and continuous overload of the anatomy trains because of swimming training (Para and able-bodied swimmers), and therefore we rejected our initial hypothesis. Those results are partially consistent with the study by Zwierzchowska et al. [24] who found that the prevalence and locations of musculoskeletal pain may be related to both to duration of disability and somatic characteristics of Para swimmers. Moreover, according to Struyf et al. [6], Kitamura et al. [7] and Matzkin [25] shoulder pain and LBP were reported as the most frequent musculoskeletal complaint in elite swimmers. Similar tendency that was found in our study corresponds with those conclusions, nevertheless in Para swimmers both duration in disability, its characteristics and etiology should also be considered as factors that could impact on the prevalence and location of musculoskeletal pain.

## Limitations

Our study has several limitations that need to be acknowledged. First of all, we investigated two groups of elite swimmers of various ages, congenital disabilities (amputees, Les Autres, cerebral palsy, blind athletes), the number of athletes (Paralympic swimming: n = 22, Olympic swimming: n = 14), and the number of females in each group (SG = 7, CG = 1). However, the study participants were elite Polish swimmers (men’s and women’s Polish national team, participants of the Olympic and Paralympic Games in Rio 2016 and Tokyo 2020) and therefore the study groups were not large. It should also be acknowledged that there are few Olympic/Paralympic swimmers at an elite level that could be included in this research. Both groups of athletes were characterized by a similar mean duration of disability and swimming training and the gender was not significant in statistical analyses for the verification of the study aim and hypothesis. Furthermore, despite of the variety of intragroup disabilities in SG, we found a similar tendency for postural disturbances, what is the strength of the present study. Future research should be focused on long-term monitoring of the training process of the athletes in order to develop and implement compensatory stimulation training programs aimed at improving the structural and functional parameters of the athletes in the context of the compensatory mechanisms that have occurred. The practical implication of this activity will be the development of a holistic exercise program for a given type of disability, regardless of sports specialization, and the preparation of original scientific publications that will allow for the provision and dissemination of global knowledge on the optimal choice of training methods and measures so that sport will be a means for the realization of the physical and mental potential of Para athletes, without interfering negatively with their health.

## Conclusions

1. This study showed that extrinsic compensatory mechanism was identified in both study groups, however only in elite Para swimmers it occurred as structural (depth of the angle of lumbar lordosis and pelvic inclination) and functional changes (ROM in the thoracic and lumbar spine) in the body posture. Moreover, the mechanism of internal compensation was identified in Para swimmers, however, contrary to our initial hypothesis, external compensation showed only partially suppressive character regarding to internal compensation.
2. The locations of the musculoskeletal complaints seems to result from both internal compensatory mechanism (Para swimmers) and continuous overload of the anatomy trains as a result of swimming training (Para and able-bodied swimmers).
3. Athletes seeking to enhance their sports performance should consider preventive strategies e.g., strength training to minimize muscular imbalance and disturbances in ROM related to swimming training.

## Data Availability

The data collected and analyzed during the current study are available from the corresponding author on reasonable request.

## Abbreviations

ROM: range of motion
BH: body height
BM: body mass
HC: hip circumference
WC: waist circumference
BMI: body mass index
BAI: body adiposity index
VFR: visceral fat rate
NMQ-7: Nordic Musculoskeletal Questionnaire for the last seven days
NMQ-6: Nordic Musculoskeletal Questionnaire for the last six months
SG1: Study group
CG: control group
kTH: Thoracic kyphosis angle in the sagittal standing
kLL: Lumbar lordosis angle in the sagittal standing
kPI: Pelvic inclination angle in the sagittal standing
SL: spinal length in the sagittal standing
SD: standard deviation
